# Leflunomide-Mediated Immunomodulation Inhibits Lesion Progression in a Vitiligo Mouse Model

**DOI:** 10.3390/ijms26146787

**Published:** 2025-07-15

**Authors:** Fang Miao, Xiaohui Li, Liang Zhao, Shijiao Zhang, Mengmeng Geng, Chuhuan Ye, Ying Shi, Tiechi Lei

**Affiliations:** Department of Dermatology, Renmin Hospital of Wuhan University, Wuhan 430060, China; fangmiao@whu.edu.cn (F.M.); xhuili@whu.edu.cn (X.L.); liangzhao1992@whu.edu.cn (L.Z.); zhangshj2023@whu.edu.cn (S.Z.); mmgeng@whu.edu.cn (M.G.); chuhuanye@whu.edu.cn (C.Y.)

**Keywords:** leflunomide, immune modulation, CD8^+^ T cell, vitiligo

## Abstract

Autoimmune CD8^+^ T cell-driven melanocyte destruction constitutes a key pathogenic mechanism in the development of vitiligo. Therefore, the pharmacological inhibition of CD8^+^ T cell effector functions and skin trafficking is a clinically viable therapeutic strategy. This study investigates leflunomide (LEF), an immunomodulatory drug with established safety in autoimmune diseases, for its therapeutic potential in a tyrosine-related protein (TRP) 2-180-induced vitiligo mouse model. Through flow cytometry, immunofluorescence, ELISA, and histopathological analyses, we systematically evaluated LEF’s effects on T cell regulation, chemokine expression, and cytokine profiles. Key findings demonstrated that LEF (20 mg/kg/day) significantly attenuated depigmentation by reducing CD8^+^ T cell infiltration and suppressing the IFN-γ-driven expression of CXCL9/10. Furthermore, LEF restored CD4^+^/CD8^+^ T cell homeostasis and rebalanced pro-inflammatory (IFN-γ, TNF-α, IL-2) and anti-inflammatory (IL-4, IL-10) cytokines, inducing a shift from Th1 to Th2. These results position LEF as an effective immunomodulator that disrupts the IFN-γ-CXCL9/10 axis and re-establishes immune balance, offering a promising repurposing strategy for halting vitiligo progression.

## 1. Introduction

Vitiligo is a chronic autoimmune disorder characterized by progressive skin depigmentation resulting from the destruction of melanocytes, affecting 0.5–2% of the global population [1,2]. Psychological consequences are severe, leading to depression, anxiety, social isolation, and perceived discrimination. Notably, these psychological impacts are independent of disease severity; even localized depigmentation on visible areas like the face or hand can provoke intense shame [3]. There are currently no FDA-approved treatments for vitiligo, and the existing treatment options are time-consuming, expensive, and of limited efficacy [4]. We sought to identify new therapeutic approaches for vitiligo, and we focused on the repurposing of existing drugs, leveraging their already established safety profiles and the potential for accelerated regulatory approval. Given the currently limited treatment options, an oral systemic therapy for vitiligo would be a valuable addition to the therapeutic arsenal.

In recent years, unprecedented progress has been made in the understanding of the pathophysiology of vitiligo. The pathological destruction of melanocytes represents the central mechanism underlying the onset and progression of vitiligo, a process driven by multiple factors, including genetic predisposition, oxidative stress, inflammatory mediator production, melanocyte detachment mechanisms, and autoimmune responses [5]. Specifically, against a background of genetic susceptibility, oxidative stress induces molecular and organelle dysfunction in melanocytes (manifested as DNA damage, lipid peroxidation, mitochondrial dysfunction, and endoplasmic reticulum stress) and triggers the exposure of melanocyte-specific antigens (e.g., Melan-A, gp100, and tyrosinase) [6]. Dysregulated innate immunity, Th1-skewed immune responses, and impaired regulatory T cell function collectively shift the immune balance toward autoimmunity, ultimately leading to CD8^+^ cytotoxic T lymphocytes (CTLs)-mediated melanocyte destruction [7]. It is widely accepted that the increased number of autoreactive, melanocyte-specific CD8^+^ T cells in the skin and blood of vitiligo patients plays a significant role in the induction and progression of the disease [8]. These specific CD8^+^ T cells are capable of producing various cytokines, including interferon-γ (IFN-γ), tumor necrosis factor (TNF-α), and granulysin, leading to the destruction of melanocytes and subsequent skin depigmentation [9]. In addition, the pro-inflammatory factor IFN-γ released by CD8^+^ T cells induces the production of chemokines, including the C-X-C motif chemokine ligands 9 and 10 (CXCL9 and CXCL10), which in turn promote the recruitment of CD8^+^ T cells to melanocytes and exacerbate the progression of vitiligo [10].

Furthermore, relatively limited advances have focused on the involvement of CD4^+^ helper T cell (Th) immunity in vitiligo. Naïve CD4^+^ T cells are known to differentiate into functionally distinct helper subsets—Th1, Th2, Th17, and regulatory T cells (Tregs)—each defined by unique cytokine profiles and transcriptional regulators [11]. In recent years, significant progress has been made in understanding the key cytokines involved in psoriasis and related autoimmune diseases. This advancement has led to the development of highly effective and well-tolerated biologics and small-molecule inhibitors, which have greatly improved patients’ quality of life [12]. However, the cytokines implicated in the pathophysiology of vitiligo differ from these. Pioneering investigations have demonstrated that subgroup cells infiltrating the perilesional skin of vitiligo exhibit a Th1-skewed phenotype, which leads to an enhanced production of inflammatory cytokines like IFN-γ and TNF-α. The imbalance of cytokine profiles is closely associated with both the severity and duration of vitiligo [13]. Targeting and correcting this imbalance presents a compelling therapeutic opportunity to effectively slow the progression of vitiligo, offering hope for improved patient outcomes.

Leflunomide (LEF) is a disease-modifying antirheumatic drug (DMARD) that inhibits the proliferation and migration of activated T cells [14]. Interestingly, a previous investigator found that 90% of 30 patients (27/30) with active non-segmental vitiligo stopped disease progression after 6 weeks of oral LEF [15]. This pilot study positions LEF as a promising disease-modifying agent for active vitiligo, with the rapid halting of progression and favorable tolerability. Building upon prior investigations, this study sought to evaluate the therapeutic efficacy of LEF in a vitiligo mouse model. Through systematic examination of LEF’s immunomodulatory properties on melanocyte-specific CD8^+^ T lymphocytes and associated inflammatory cytokine networks, we aimed to elucidate both the therapeutic potential and underlying molecular mechanisms of LEF as a novel treatment candidate for vitiligo.

## 2. Results

### 2.1. LEF Ameliorates Progressive Depigmentation in a Vitiligo Mouse Model

**Figure 1:**
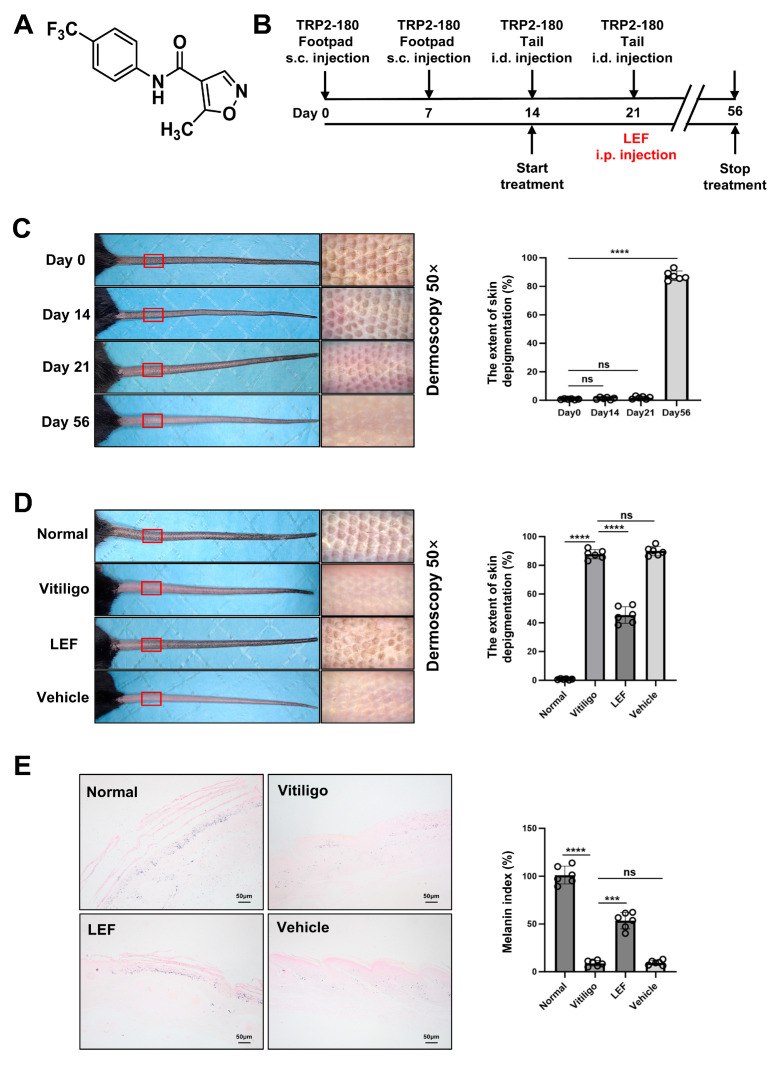
Leflunomide (LEF) ameliorates progressive depigmentation in a mouse model of vitiligo. (**A**) Structure of LEF. (**B**) Schematic diagram of vitiligo induction and treatment. C57BL/6 mice were immunized subcutaneously (s.c.) into the footpad with tyrosinase-related protein (TRP) 2-180 on days 0 and 7, followed by intradermal (i.d.) boost immunization in the tail skin on days 14 and 21. LEF was intraperitoneally (i.p.) injected from day 14 to day 56, twice weekly (red mark). Mice were euthanized on day 56 for subsequent experiments. (**C**) Longitudinal monitoring of depigmented lesions on murine tails post-TRP2-180 immunization. Representative photographs (left), corresponding dermoscopic images (middle; red-boxed areas), and quantification of depigmentation areas (right). (**D**) Representative images of tail depigmentation 5 weeks post-final immunization (left), dermoscopic features (middle; red-boxed areas), and quantification of depigmentation areas (right) are shown. (**E**) Representative pictures and the bar chart showing Fontana–Masson staining in the tail sections (scale bar = 50 μm). Data are representative of three independent experiments (*n* = 6 mice/group). Values are mean ± SD. *** *p* < 0.001, **** *p* < 0.0001, ns, not significant. *p* values were determined by ordinary one-way ANOVA. SD: standard deviation.

The chemical structure of LEF is depicted in Figure 1A. A vitiligo mouse model, constructed according to the methodological protocols described in the Materials and Methods, was employed to evaluate the therapeutic potential of LEF (Figure 1B). To induce melanocyte-specific autoreactive CD8^+^ T lymphocytes, mice were immunized repetitively via hind footpad injection with TRP2-180. At 8 weeks post-initial immunization (5 weeks following the final boost), well-established vitiligo lesions were observed in the tail skin of immunized animals, with the depigmented area accounting for 87.70 ± 3.20% of the total tail area (Figure 1C). Quantitative analysis of Fontana–Masson staining revealed a reduction in melanin content (melanin index: 8.93 ± 2.91% [vitiligo model group] vs. 101.10 ± 9.30% [normal controls]; *p* < 0.0001; Figure 1E), closely recapitulating human vitiligo pathology. LEF treatment, initiated on day 14 via twice-weekly intraperitoneal injections, significantly attenuated lesion progression: the depigmented area was reduced to 45.30 ± 5.82% in LEF-treated mice, compared to 87.70 ± 3.20% in the vitiligo model group (Figure 1D). Fontana–Masson staining further confirmed that LEF-treated mice retained higher melanin deposition than the vitiligo model group (melanin index: 53.52 ± 8.53% vs. 8.93 ± 2.91%; *p* < 0.001; Figure 1E). Meanwhile, no significant differences were observed in the depigmented area or melanin deposition between the vitiligo model group and the vehicle control group.

### 2.2. LEF Suppresses Cutaneous CD8^+^ T Lymphocyte Infiltration

**Figure 2:**
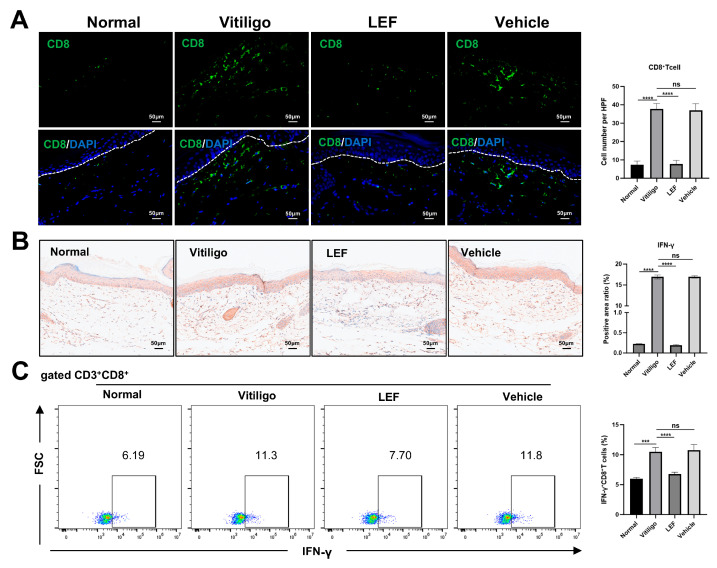
Leflunomide (LEF) inhibits CD8^+^ T cell infiltration and IFN-γ production in a mouse model of vitiligo. (**A**) Representative immunofluorescent images (left) and quantification (right) of CD8^+^ T cells in the mouse tail sections. (**B**) Representative immunohistochemistry images (left) and quantifications (right) of IFN-γ in the tail skin lesions. (**C**) Flow cytometric analysis of the percentage of IFN-γ^+^ CD8^+^ T cells in the peripheral blood mononuclear cells (PBMCs) of the mice, representative examples (left), and frequency analysis (right). All data were collected on day 56 after initial immunization. Values are mean ± SD. *** *p* < 0.001, **** *p* < 0.0001, ns, not significant. *p* values were determined by ordinary one-way ANOVA. HPF: high-power field; IFN-γ: interferon-γ; SD: standard deviation.

The activation of autoreactive CD8^+^ T lymphocytes targeting melanocytes, along with their directed migration to the skin, drives the progression, exacerbation, and recurrence of vitiligo. Immunofluorescence revealed that vitiligo mice exhibited a significant elevation in CD8^+^ T cell numbers (37.67 ± 3.05 cells/high-power field [HPF]) compared to normal controls (7.33 ± 2.08 cells/HPF; *p* < 0.0001; Figure 2A). Notably, LEF treatment significantly reduced CD8^+^ T cell counts to 7.66 ± 2.08 cells/HPF, compared to the vitiligo model group (37.67 ± 3.05 cells/HPF; *p* < 0.0001; Figure 2A), underscoring its capacity for the targeted inhibition of pathogenic CD8^+^ T cell recruitment. Histologic quantification further demonstrated that LEF administration significantly suppressed IFN-γ expression levels in lesional skin: The vitiligo model mice exhibited an IFN-γ-positive area ratio of 16.92 ± 0.46%, whereas LEF-treated mice showed only 0.19 ± 0.1% (*p* < 0.0001; Figure 2B). Finally, the flow cytometric analysis of peripheral blood mononuclear cells (PBMCs) revealed a marked decrease in the frequency of IFN-γ^+^ CD8^+^ T cells after in vivo LEF treatment: 10.47 ± 0.72% of the IFN-γ^+^ CD8^+^ T cell population in the vitiligo model group compared to only 6.76 ± 0.30% in LEF-treated mice (*p* < 0.0001; Figure 2C, Supplementary Figure S1).

### 2.3. LEF Downregulates CXCL9 and 10 Chemokine Expression

**Figure 3:**
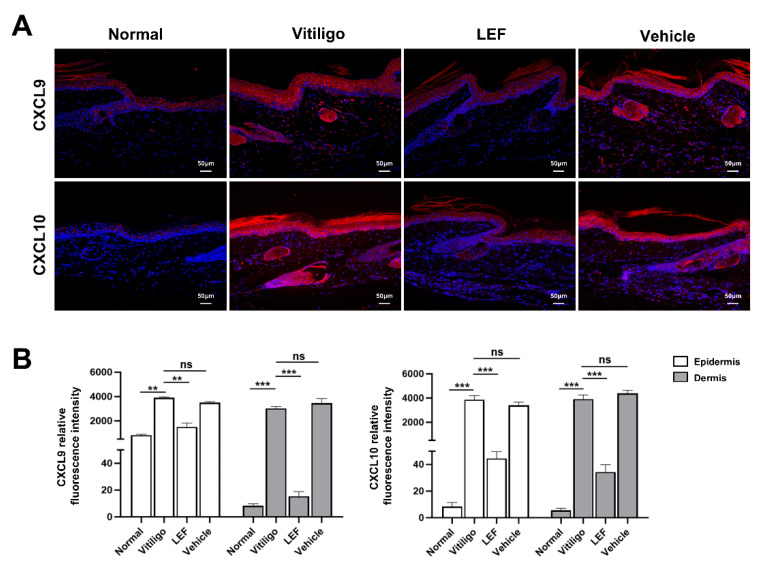
Leflunomide (LEF) downregulates C-X-C motif chemokine ligands 9 and 10 (CXCL9/10) chemokine expression in a mouse model of vitiligo. (**A**) Immunofluorescence staining of CXCL9/10, scale bar = 50 μm. (**B**) Quantification of CXCL9/10 staining area in epidermis and dermis separately. Values are mean ± SD. ** *p* < 0.01, *** *p* < 0.001, ns, not significant. *p* values were determined by one-way ANOVA. SD: standard deviation.

Previous studies have shown that various chemokines are essential for the development of vitiligo, especially CXCL9 and CXCL10. CXCL9 primarily facilitates the recruitment of CTLs to peripheral tissues, while CXCL10 drives their migration and precise localization near melanocyte clusters. To elucidate whether LEF affects the expression of CXCL9 and CXCL10, immunofluorescent staining was used to illuminate these two chemokines. As shown in Figure 3A,B, normal skin tissues exhibited minimal CXCL9 and 10 expression: CXCL9 displayed low mean fluorescence intensity (MFI) in the epidermis (819.30 ± 83.61) and negligible MFI in the dermis (8.33 ± 1.52), while CXCL10 MFI was nearly undetectable in both the epidermis (8.33 ± 3.05) and dermis (5.66 ± 1.52). In contrast, vitiligo model mice showed a marked upregulation of CXCL9 and 10: CXCL9 MFI increased to 3910 ± 62.39 (epidermis) and 3018 ± 157.8 (dermis), and CXCL10 MFI similarly rose to 3868 ± 331.9 (epidermis) and 3927 ± 329 (dermis) relative to normal controls. Notably, LEF treatment significantly reduced these levels: CXCL9 MFI decreased to 1498 ± 324.1 (epidermis) and 15.33 ± 3.51 (dermis), whereas CXCL10 MFI declined more significantly to 44.67 ± 5.13 (epidermis) and 34.33 ± 5.50 (dermis).

### 2.4. LEF Restores CD4^+^/CD8^+^ T Lymphocytes Homeostasis

**Figure 4:**
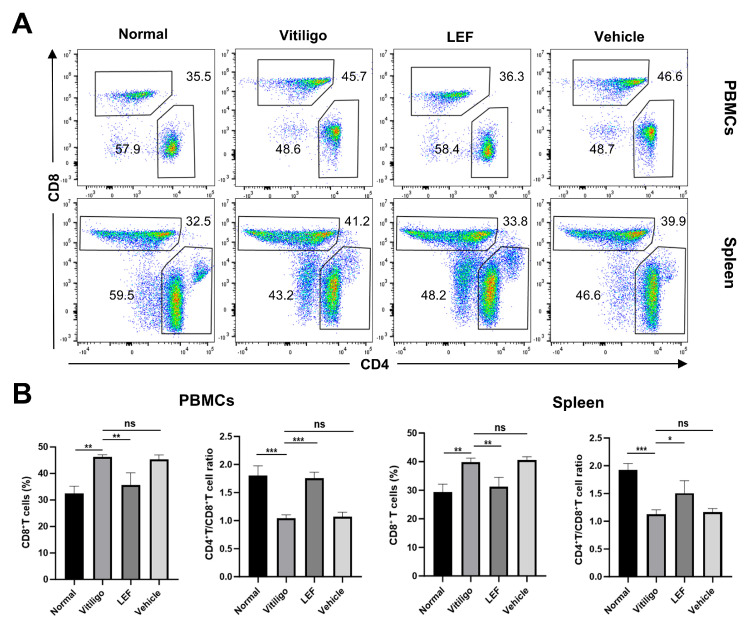
Leflunomide (LEF) restores CD4^+^/CD8^+^ T lymphocyte homeostasis. (**A**) Representative flow cytometry plot of the percentage of CD8^+^ T cells and CD4^+^ T cells among them in peripheral blood mononuclear cells (PBMCs; top) and spleen (bottom) of mice. (**B**) Bar chart showing CD8^+^ T cell frequencies (left) and CD4^+^/CD8^+^ ratio (right) in PBMCs and spleen. All data were collected on day 56 after initial immunization. Values are mean ± SD. * *p* < 0.05, ** *p* < 0.01, *** *p* < 0.001, ns, not significant. *p* values were determined by one-way ANOVA. SD: standard deviation.

To examine whether LEF modulates the CD4^+^/CD8^+^ T cell balance, we isolated spleen and PBMCs from mice and analyzed them via flow cytometry (Figure 4A, Supplementary Figure S2). Initially, the flow cytometry analysis of PBMCs revealed a significant decrease in the CD4^+^/CD8^+^ T cell ratio in vitiligo mice compared to the normal group (1.04 ± 0.05 vs. 1.79 ± 0.17; *p* < 0.001). This reduction was attributed to an increase in the proportion of CD8^+^ T cells (32.43 ± 2.70% [normal controls] vs. 46.27 ± 0.81% [vitiligo model group]; *p* < 0.01). Following LEF treatment, the percentage of CD8^+^ T cells in mouse PBMCs decreased to 35.67 ± 4.58%, accompanied by an elevation in the CD4^+^/CD8^+^ T cell ratio to 1.75 ± 0.10. Notably, the flow cytometric analysis of splenocytes in mice showed a consistent trend with the PBMCs results (Figure 4B).

### 2.5. LEF Modulates Pro-Inflammatory and Anti-Inflammatory Cytokine Profiles

**Figure 5:**
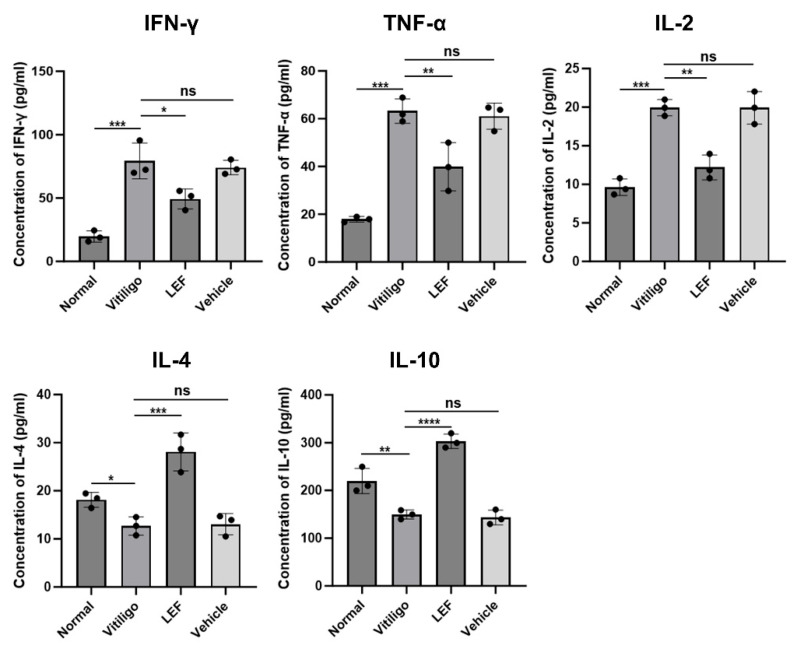
Leflunomide (LEF) rebalances pro- and anti-inflammatory cytokine networks. Serum levels of IFN-γ, TNF-α, IL-2, IL-4, and IL-10 were detected by ELISA. Values are mean ± SD. * *p* < 0.05, ** *p* < 0.01, *** *p* < 0.001, **** *p* < 0.0001, ns, not significant. *p* values were determined by one-way ANOVA. SD: standard deviation.

Cytokine dysregulation is central to vitiligo pathogenesis, driving immune dysregulation and disease progression through its dual effects on inflammatory responses and melanocyte survival. To evaluate the immunomodulatory effects of LEF, serum levels of pro-inflammatory cytokines (IFN-γ, TNF-α, Interleukin [IL]-2) and anti-inflammatory cytokines (IL-4, IL-10) were quantified. In vitiligo mice, IFN-γ, TNF-α, and IL-2 levels were significantly elevated compared to normal controls (*p* < 0.001 for all cytokines). LEF treatment normalized these profiles, reducing IFN-γ, TNF-α, and IL-2 by 1.6-fold (*p* = 0.013), 1.5-fold (*p* = 0.008), and 1.6-fold (*p* = 0.001), respectively, while elevating IL-4 and IL-10 by 2.2-fold (*p* = 0.0004) and 2.0-fold (*p* = 0.0001) (Figure 5). This dual modulation of cytokine signaling suggests that LEF promotes a Th1-to-Th2 shift, countering the inflammatory milieu that perpetuates melanocyte destruction.

## 3. Discussion

Current therapeutic options for vitiligo remain limited in terms of efficacy, cost-effectiveness, and convenience [16]. This study elucidates the therapeutic potential of LEF in improving depigmentation and modulating immune dysregulation in a vitiligo mouse model. Our findings indicate that LEF administration can alleviate progressive pigmentation, inhibit autoreactive CD8^+^ T cell infiltration in lesions, and downregulate the expression of CXCL9/10 chemokines. Additionally, LEF restored the CD4^+^/CD8^+^ T cell ratio and modulated the balance between pro- and anti-inflammatory cytokines, inducing a shift from Th1- to Th2-type immune responses. These results collectively suggest that LEF exerts multifaceted immunomodulatory effects, positioning it as a promising candidate for vitiligo therapy.

LEF exerts its therapeutic effects by inhibiting dihydroorotate dehydrogenase (DHODH), a key enzyme in the de novo pyrimidine biosynthesis pathway. By targeting DHODH, LEF depletes intracellular pyrimidine pools, thereby suppressing the proliferation of activated T and B lymphocytes implicated in autoimmune pathogenesis [17]. Given the established safety profile of LEF in treating inflammatory-mediated autoimmune diseases, repurposing LEF for vitiligo therapy holds translational promise [18]. Notably, in a pilot study, Awad et al. reported that 90% of vitiligo patients subjected to LEF treatment showed a cessation of disease progression, supporting the translational potential of LEF [15]. While human trials provide critical clinical validation, animal experiments are essential for dissecting the precise immunomodulatory mechanisms that underpin LEF’s efficacy, which is difficult to isolate in human subjects due to inter-individual variability and ethical constraints. Our insight into the LEF’s mechanism of action—targeting both immune cell dynamics and inflammatory signaling—provides a theoretical basis for clinical trials.

Vitiligo pathogenesis arises from the interplay of multiple factors, including genetic predisposition, melanocyte vulnerability to oxidative stress, metabolic dysregulation, and autoimmune cytotoxicity. Firstly, genome-wide association studies (GWAS) implicate more than 50 risk loci, which disrupt immune tolerance, melanocyte function, and inflammasome activation [19,20,21]. In addition, intrinsic defects in melanocytes can exacerbate oxidative damage. Mitochondrial dysfunction leads to the excessive production of reactive oxygen species (ROS), while impaired antioxidant capacity in melanocytes (e.g., catalase deficiency) triggers endoplasmic reticulum stress and apoptosis. Moreover, emerging evidence underscores that systemic metabolic abnormalities are integral to the pathogenesis of vitiligo, beyond associations to drive disease progression through interconnected pathways of oxidative stress, inflammation, and immune dysregulation. These investigations further reveal pervasive metabolic reprogramming across peripheral blood, lesional skin, and gut microbiota environments in both vitiligo patients and murine models, characterized by alterations in metabolic enzymes, upstream signaling molecules, and downstream metabolites [22]. Specifically, patients with vitiligo exhibit significant dyslipidemia, characterized by elevated low-density lipoprotein (LDL) levels and reduced high-density lipoprotein (HDL) levels, which not only reflects metabolic syndrome comorbidity but also contributes to melanocyte damage via increased oxidative stress and lipid peroxidation. Additionally, deficiencies in micronutrients such as folate and vitamin D exacerbate this vulnerability; folate deficiency elevates homocysteine levels, leading to increased oxidative damage and endothelial dysfunction, while vitamin D insufficiency impairs immune modulation, fostering an autoimmune milieu against melanocytes. These alterations are further compounded by broader metabolic disruptions, including insulin resistance, dysregulated amino acid metabolism, glucose metabolism, and lipid metabolism, which collectively lead to melanocyte destruction and drive the progression of vitiligo [23]. Furthermore, emerging evidence has demonstrated the presence of abundant autoreactive CD8^+^ T cells targeting melanocytes in both the skin and peripheral blood of vitiligo patients. These melanocyte-specific cytotoxic CD8^+^ T cells release effector molecules (including IFN-γ, granzyme B, and perforin), and are critical for melanocyte destruction and the depigmentation of the skin [24].

To investigate the potential therapeutic effects of LEF in vitiligo and elucidate its underlying mechanisms, we employed the method reported by You et al. to establish a vitiligo mouse model [25]. Confirmed by melanin staining, epidermal decolorization was observed in the tail skin of the developing mouse model, with disease characteristics similar to human vitiligo lesions. Additionally, reduced levels of CD8^+^ T lymphocyte infiltration were found in skin lesions of LEF-treated mice with vitiligo. Our findings indicate that LEF effectively prevents pigment loss in the vitiligo mouse model by blocking melanocyte destruction via the suppression of CD8^+^ T cell-mediated cytotoxic responses (Figure 1 and Figure 2).

**Figure 6:**
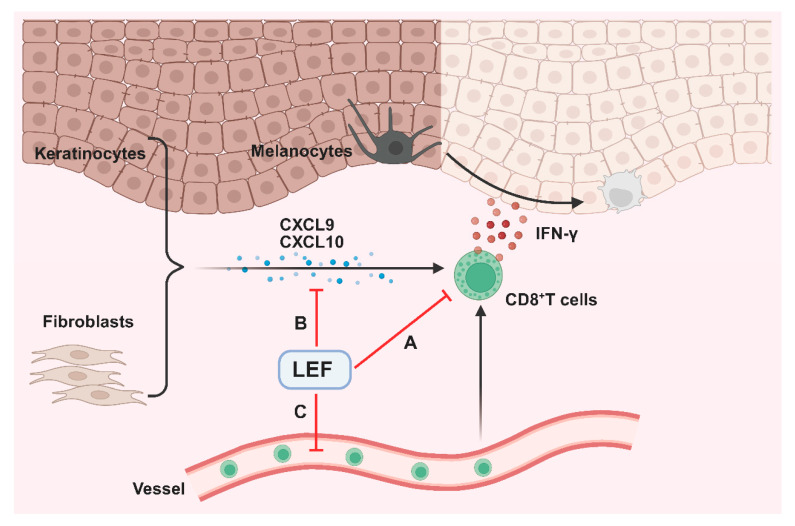
Leflunomide (LEF) ameliorated the depigmentation of vitiligo. Elevated levels of interferon gamma (IFN-γ) in vitiligo skin induce the production of C-X-C motif chemokine ligands 9 and 10 (CXCL9 and CXCL10), which further recruit circulating autoreactive CD8^+^ T cells. Autoreactive CD8^+^ T cells destroy melanocytes and establish a high IFN-γ profile. LEF: (A) Suppresses CD8^+^ T cells infiltration and effector function; (B) reduces CXCL9/10 production in keratinocytes and fibroblasts; (C) blocks CD8^+^ T cells migration and rescues the loss of melanocytes.

In addition, as an autoimmune disease, vitiligo involves complex interactions of multiple cytokines like interferons, tumor necrosis factor, and chemokines [26]. Elevated levels of IFN-γ induce the production of CXCL9 and CXCL10, which share the common receptor C-X-C chemokine receptor type 3 (CXCR3) on CD8^+^ T cells, to form the IFN-γ-CXCL9/10-CXCR3 (ICC) signaling axis (Figure 6). This axis plays a pivotal role in promoting the intradermal infiltration of melanocyte-specific CD8^+^ T lymphocytes and driving the progressive depigmentation process in vitiligo pathogenesis [27]. Multiple clinical studies have demonstrated significantly elevated serum and tissue levels of CXCL9/10 in patients with active vitiligo compared to healthy controls [28]. The therapeutic potential of targeting this axis is highlighted by previous studies demonstrating that neutralization of CXCL10 not only prevents but also reverses depigmentation in murine vitiligo models [29]. These findings position the ICC axis as both a key pathomechanistic driver and a promising therapeutic target in vitiligo [30]. Notably, our study established a marked reduction in CXCL9/10 expression following LEF treatment, underscoring a novel dimension of its immunomodulatory activity in vitiligo. This suppression of CXCL9/10 by LEF suggests interference with upstream signaling pathways, potentially involving IFN-γ. Future studies should clarify whether LEF directly inhibits chemokine production in keratinocytes or acts indirectly through the modulation of immune cells.

Emerging evidence implicates the dysregulation of cellular immunity, particularly perturbations in CD4^+^ and CD8^+^ T lymphocytes, as a critical driver in the immunopathogenesis of vitiligo [31]. The CD4^+^/CD8^+^ T cell ratio is decreased in progressive vitiligo, and successful repigmentation is accompanied by an elevated CD4^+^/CD8^+^ T cell ratio of the lesions [32]. In our study, LEF restored the skewed CD4^+^/CD8^+^ ratio, partly due to the selective inhibition of CD8^+^ T cell expansion and the preservation of CD4^+^ T cell subsets (Figure 4). In addition, a variety of cytokines secreted by T helper cell subsets- Th1 (IFN-γ, TNF-α, IL-2) and Th2 (IL-4, IL-10, IL-13) not only exacerbate autoimmune responses but also perpetuate the vicious cycle of immune dysfunction, ultimately leading to the clinical manifestations of vitiligo [33]. Concurrently, the cytokine profile shift—characterized by reduced IFN-γ, TNF-α, and IL-2, alongside elevated IL-4 and IL-10—suggests that LEF promotes an anti-inflammatory milieu, countering the Th1 axis that drives vitiligo (Figure 5). Grimes et al. reported that IFN-γ and TNF-α were elevated both in the skin lesions and the serum in the case of vitiligo patients [34]. IFN-γ is a hallmark cytokine in vitiligo, which is predominantly secreted by activated CD8^+^ T cells and Th1 lymphocytes [35,36]. TNF-α promotes the differentiation of cytotoxic CD8^+^ T lymphocytes, thereby enhancing IFN-γ secretion [37]. In addition, previous studies have shown that serum IL-2 levels are significantly elevated in patients with localized and generalized vitiligo compared to healthy controls [38,39]. Yeo et al. conducted a study involving 79 vitiligo patients, measuring serum soluble IL-2R via ELISA, and reported similar findings. In their study, serum IL-2 level was correlated with disease duration and activity [40,41]. IL-4 is a Th2-associated cytokine, which is reduced in vitiligo patients, contributing to Th1/Th2 imbalance [42]. Notably, IL-10 maintains immune tolerance through dual mechanisms: the direct suppression of autoreactive CD8^+^ T cells and the potentiation of Treg-mediated immunomodulation. Vitiligo has been pathologically characterized as a Th1-polarized disorder [38,43], and our findings provide novel therapeutic insights through cytokine rebalancing. LEF administration demonstrated significant immunomodulatory capacity, reducing the serum levels of Th1-associated pro-inflammatory cytokines (IFN-γ, TNF-α, IL-2) while upregulating Th2-derived anti-inflammatory mediators (IL-4, IL-10) in vitiligo mice.

The present study provides valuable insights into the immunomodulatory effects of LEF in vitiligo, yet it is important to acknowledge its inherent limitations. Vitiligo is a multifactorial disorder driven by intricate interactions between immune, oxidative, metabolic, and genetic mechanisms. Our work focused on LEF’s regulation of CD8^+^ T cell effector functions, IFN-γ/CXCL9/10 signaling, and T helper cell balance. However, the potential non-immune modulatory role of LEF in these processes remains unaddressed. Another limitation is that the current study was conducted solely in animal models; large-scale, long-term clinical trials involving diverse patient populations and extended follow-up periods are essential to evaluate LEF’s efficacy and safety in human vitiligo comprehensively.

In summary, our results suggest that LEF inhibits the secretion of chemokines, thereby attenuating the migration and intradermal infiltration of CD8^+^ T cells and suppressing the progressive depigmentation of skin lesions in a vitiligo mouse model. Additionally, LEF modulates the balance of CD4^+^ and CD8^+^ T lymphocytes and lowers the serum levels of pro-inflammatory cytokines. These insights deepen our understanding of the therapeutic potential of LEF beyond its current application and offer a new referential option for clinical dosing of vitiligo patients. Further mechanistic exploration will refine its application and uncover novel targets within the complex immunopathology of vitiligo.

## 4. Materials and Methods

### 4.1. Animals and Experimental Design

Male C57BL/6 mice (4 weeks old) were randomly divided into four groups (*n* = 6 per group): (1) Group 1—normal control: no immunization or treatment; (2) Group 2—vitiligo model: immunized with TRP2-180 (sequence: SVYDFFVWL; Sangon Biotech, Shanghai, China) with lipopolysaccharide (LPS; Invitrogen, Carlsbad, CA, USA) and CpG oligonucleotides 1826 (CpG-ODN 1826; Sangon Biotech); (3) Group 3—LEF-treated model: immunized as above, treated with LEF (Sangon Biotech) dissolved in 0.5% (*w*/*v*) carboxymethylcellulose sodium (CMC-Na; Sangon Biotech) post-second immunization; (4) Group 4—vehicle control: immunized as above, administered equivalent volumes of 0.5% (*w*/*v*) CMC-Na on the same schedule. Mice were maintained in specific pathogen-free (SPF) facilities with controlled conditions: 12 h light/dark cycle, 22 ± 1 °C ambient temperature, 50–60% relative humidity, and ad libitum access to food and water. All animal protocols were approved by the Ethics Committee of the Renmin Hospital of Wuhan University (Approval No. WDRM2021-KS075) and adhered to established animal guidelines.

### 4.2. Establishment of a Vitiligo Mouse Model and LEF Treatment

Vitiligo was induced in Groups 2–4 following an established protocol [25]. Briefly, 4-week-old male C57BL/6 mice (Shulaibao Biotechnology, Wuhan, China) were twice immunized with TRP2-180 (50 µg), LPS (5 µg), and CpG-ODN 1826 (5 µg) in the hind footpad after a time interval of one week. Similarly, at a week’s interval, the next two injections of similar doses were given intradermally in the tails of mice. Group 3 mice received twice-weekly intraperitoneal injections of LEF (20 mg/kg body weight; Sangon Biotech) dissolved in 0.5% (*w*/*v*) CMC-Na (Sangon Biotech) from week 2 post-initial immunization until euthanasia at week 8 [14]. Simultaneously, Group 4 was administered equivalent volumes of 0.5% CMC-Na following the same regimen. Normal control: mice were not immunized with anything. The degree of depigmentation was quantified objectively by calculating the percentage of the depigmented area relative to the total dorsal tail surface area using ImageJ software (NIH, v1.53).

### 4.3. Fontana Masson Staining

Fontana–Masson staining was performed on 4 μm paraffin sections to detect melanin. Sections were baked (60 °C, 30 min), deparaffinized, and rehydrated. After deionized water rinsing, slides were incubated in silver–ammonia solution for 12–18 h at 25 °C under light protection. Following sequential rinses and 0.2% gold chloride toning (8 min), unbound silver was removed with 5% sodium thiosulfate (10 min). After washing again with distilled water, sections were counterstained with Ponceau for 3 min, then rinsed in distilled water, dehydrated thoroughly and rapidly in absolute alcohol, cleared, and mounted. Melanin granules were identified as jet-black punctate deposits (0.5–2 μm) via a brightfield microscope (Olympus, Tokyo, Japan), and the grayscale value of the epidermis was calculated.

### 4.4. Immunohistochemical and Immunofluorescence Staining

Paraffin-embedded mouse tail skin sections (4 μm) were dewaxed in xylene and rehydrated through a graded ethanol series. Antigen retrieval was performed by boiling in 10 mM citrate buffer (pH 6.0, 95 °C, 20 min). Endogenous peroxidase activity was quenched with 3% H_2_O_2_ for 15 min. Sections were blocked with 10% normal goat serum for 1 h at 25 °C, then incubated overnight at 4 °C with anti-IFN-γ (1:200 dilution, DF6045, Affinity). After PBS washes, slides were treated with HRP-conjugated goat anti-rabbit IgG (1:2000 dilution, ab205718, Abcam) for 1 h at 25 °C. The signal was developed using 3,3′-diaminobenzidine (DAB; DAB4033, Maxim Biotech, Fuzhou, China) for 2 min, followed by Mayer’s hematoxylin counterstaining (30 s). For immunofluorescence staining, the tissue sections were incubated with the following primary antibodies: anti-CD8 (1:700 dilution, GB114196, Servicebio, Wuhan, China), anti-CXCL9 (1:200 dilution, 701117, Invitrogen), and anti-CXCL10 (1:200 dilution, 701225, Invitrogen), and were then incubated with species-specific secondary antibodies, Cy3-conjugated goat anti-rabbit IgG (1:200 dilution, GB21303, Servicebio), or FITC-conjugated goat anti-rabbit IgG (1:200 dilution, GB22303, Servicebio), for 1 h at 25 °C. Nuclei were stained using DAPI solution. Imaging was performed using a fluorescence microscope (Olympus, Tokyo, Japan). DAB^+^ areas and fluorescence intensity were quantified using ImageJ software.

### 4.5. Flow Cytometry

All mice were euthanized on day 56 (corresponding to 8 weeks post-initial immunization and 6 weeks post-initiation of LEF treatment), and peripheral blood along with spleens was harvested for flow cytometric analysis. Peripheral blood (125 μL) was collected from mice via retro-orbital bleeding into EDTA-coated tubes (QX0002, Servicebio). Spleens were mechanically dissociated using sterile frosted slides, and single-cell suspensions were filtered through 70 μm nylon mesh strainers (W15-1070, Servicebio). Erythrocytes were lysed using ACK lysing buffer (G2015, Servicebio) for 5 min at 25 °C, followed by two washes in PBS containing 2% FBS (staining buffer). Cells were harvested for analysis, and a cocktail of Brefeldin A and Monensin (CS1002, Multi Sciences, Hangzhou, China) was added to suppress cytokine release at 6 h before intracellular cytokine staining. For surface marker staining, cells were preincubated with Mouse Fc Blocking Solution (abs9477, Absin, Shanghai, China) for 10 min at 25 °C to minimize nonspecific binding. Subsequent surface staining utilized the following antibody panel (25 μL total volume per 10^6^ cells): anti-mouse FITC-conjugated CD3 (F2100301, Multi Sciences), PE-conjugated CD8 (F2100802, Multi Sciences), and PerCP-Cy5.5-conjugated CD4 (F2100404, Multi Sciences). Cells were incubated with antibody cocktails for 30 min at 25 °C protected from light. After surface staining, cells were fixed and permeabilized using the Fix/Perm Kit (Multi Sciences, CS1002) following the manufacturer’s guidelines. Intracellular IFN-γ was detected with PE-Cy7-conjugated anti-IFN-γ (1:50, F21IFNG05, Multi Sciences) in perm/wash buffer for 1 h at 25 °C. After two washes in the staining buffer, the samples were suspended and analyzed with flow cytometry (Beckman Coulter, Miami, FL, USA).

### 4.6. Enzyme-Linked Immunosorbent Assay (ELISA)

Whole blood was collected in clot activator tubes (QX0024, Servicebio) and allowed to clot at 25 °C for 30 min. The serum was separated by centrifugation at 1000× *g* for 10 min. The upper layer of the serum was isolated and stored in cryovials at −80 °C till cytokine analysis. ELISA kits for the chemokines IFN-γ (EK280, Multi Sciences), TNF-α (EK282, Multi Sciences), IL-2 (EK202, Multi Sciences), IL-4 (EK204, Multi Sciences), and IL-10 (EK210, Multi Sciences) were employed to measure their concentrations in serum, following the instructions provided by the manufacturers.

### 4.7. Statistical Analysis

Each experiment was repeated at least three times, and the resulting data were analyzed using an unpaired, two-tailed Student’s *t*-test or a one-way analysis of variance (ANOVA). The statistical analyses were conducted with GraphPad Prism software (version 7.0, GraphPad Software, Inc., San Diego, CA, USA). All data are presented as means ± standard deviation (SD). Statistical significance was determined at *p* < 0.05.

## Data Availability

Data supporting the results of this study are available from the corresponding author upon reasonable request.

## Supplementary Materials

The following supporting information can be downloaded at https://www.mdpi.com/article/10.3390/ijms26146787/s1.

## Abbreviations

LEF	Leflunomide
TRP2-180	Tyrosine-related protein 2 -180
IFN-γ	Interferon-γ
TNF-α	Tumor necrosis factor-α
CXCL9	C-X-C motif chemokine ligand 9
CXCL10	C-X-C motif chemokine ligand 10
CXCR3	C-X-C chemokine receptor type 3
PBMCs	Peripheral blood mononuclear cells
CTLs	Cytotoxic T lymphocytes
IL-2	Interleukin-2
IL-4	Interleukin-4
IL-10	Interleukin-10
ROS	Reactive oxygen species
