# Supplementary material for: Leflunomide-Mediated Immunomodulation Inhibits Lesion Progression in a Vitiligo Mouse Model
Source: Int J Mol Sci. 2025 Jul 15;26(14):6787. doi: 10.3390/ijms26146787 (PMC12296137; doi:10.3390/ijms26146787)
Supplement: Supplementary file 1 [file ijms-26-06787-s001.zip › ijms-3628473-supplementary.pdf]

Supplementary Figures and Figure Legends

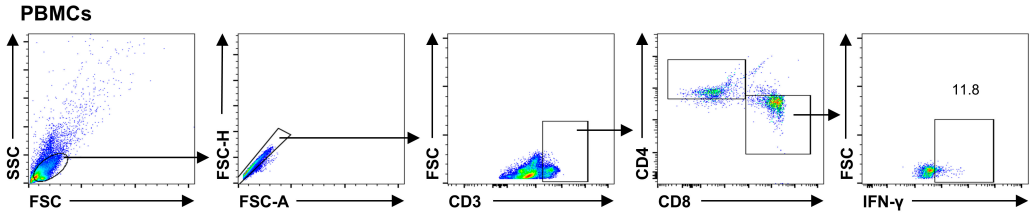

Supplementary Figure S1: Flow cytometric sorting strategy for immune cells from mouse peripheral blood mononuclear cells (PBMCs).

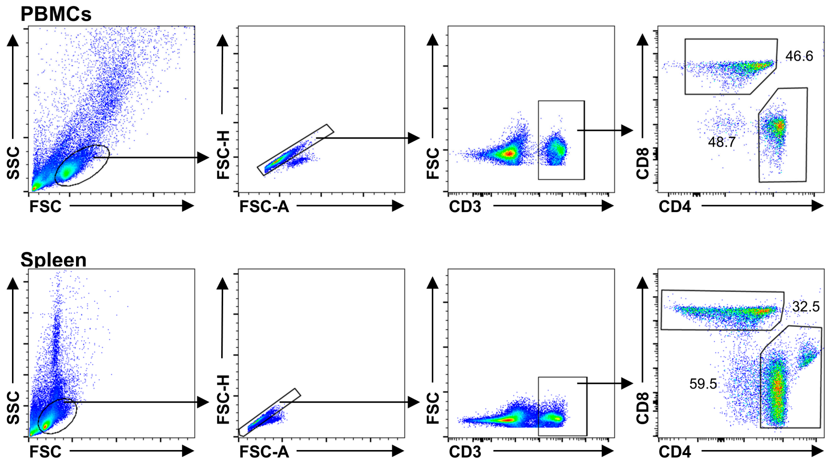

Supplementary Figure S2: Flow cytometric sorting strategy for IFN-γ^{+}CD8^{+} T cells from mouse peripheral blood mononuclear cells (PBMCs) and spleen.
